# Identifying a sublingual triangle as the ideal site for assessment of sublingual microcirculation

**DOI:** 10.1007/s10877-022-00936-9

**Published:** 2022-11-10

**Authors:** Zühre Uz, Olcay Dilken, Dan M. J. Milstein, Matthias Peter Hilty, David de Haan, Yasin Ince, Lucinda Shen, Julia Houtzager, Lotte C. Franken, Thomas M. van Gulik, Can Ince

**Affiliations:** 1 Department of Translational Physiology, Location: AMC, Amsterdam University Medical Centre (UMC), University of Amsterdam, Amsterdam, The Netherlands; 2 Department of Surgery, Location: AMC, Amsterdam UMC, University of Amsterdam, Amsterdam, The Netherlands; 3 Department of Intensive Care, Erasmus MC, University Medical Centre, Rotterdam, the Netherlands; 4 Department of Oral & Maxillofacial Surgery, Location: AMC, Amsterdam UMC, University of Amsterdam, Amsterdam, The Netherlands; 5 Department of Oral Medicine, Academic Centre for Dentistry Amsterdam (ACTA), University of Amsterdam and VU University Amsterdam, Amsterdam, The Netherlands

**Keywords:** IDF imaging, Microcirculation, Sublingual mucosa

## Abstract

The sublingual mucosa is a commonly used intraoral location for identifying microcirculatory alterations using handheld vital microscopes (HVMs). The anatomic description of the sublingual cave and its related training have not been adequately introduced. The aim of this study was to introduce anatomy guided sublingual microcirculatory assessment. Measurements were acquired from the floor of the mouth using incident dark-field (IDF) imaging before (T0) and after (T1) sublingual cave anatomy instructed training. Instructions consists of examining a specific region of interested identified through observable anatomical structures adjacent and bilaterally to the lingual frenulum which is next to the sublingual papilla. The anatomical location called the sublingual triangle, was identified as stationed between the lingual frenulum, the sublingual fold and ventrally to the tongue. Small, large, and total vessel density datasets (SVD, LVD and TVD respectively) obtained by non-instructed and instructed measurements (NIN (T0) and IM (T1) respectively) were compared. Microvascular structures were analyzed, and the presence of salivary duct-related microcirculation was identified. A total of 72 video clips were used for analysis in which TVD, but not LVD and SVD, was higher in IM compared to NIM (NIM vs. IM, 25 ± 2 vs. 27 ± 3 mm/mm^2^ (p = 0.044), LVD NIM vs. IM: 7 ± 1 vs. 8 ± 1mm/mm^2^ (p = 0.092), SVD NIM vs. IM: 18 ± 2 vs. 20 ± 3 mm/mm^2^ (p = 0.103)). IM resulted in microcirculatory assessments which included morphological properties such as capillaries, venules and arterioles, without salivary duct-associated microcirculation. The sublingual triangle identified in this study showed consistent network-based microcirculation, without interference from microcirculation associated with specialized anatomic structures. These findings suggest that the sublingual triangle, an anatomy guided location, yielded sublingual based measurements that conforms with international guidelines. IM showed higher TVD values, and future studies are needed with larger sample sizes to prove differences in microcirculatory parameters.

## Introduction

The sublingual mucosa, specifically the sublingual region, presents the mostly frequently chosen anatomical location to perform microcirculatory evaluation using handheld vital microscopes. [1–3] [4–12]. This anatomical region is easily accessible which offers opportunities to quickly obtain microcirculatory video images for assessment, an ideal non-invasive strategy to obtain sublingual measurements at the bed side [13]. The microcirculation is a collective network of microvasculature differing in diameter and vessel wall thicknesses. Capillaries are mainly responsible for the exchange of diffusive gases and are on average typically < 10 μm in diameter [1]. Venules have thinner vessel walls and are generally pressure sensitive structures which can succumb to pressure artefacts owing of their thinner wall structure and lower transmural pressures. Vein susceptibility to pressure artefacts can be used advantageously to generate high-quality microcirculatory video images that are viable for clinical analysis [7]. Therefore, the region of interest for targeting microcirculatory imaging should include a distribution of capillaries, small venules and large venules in the field of view.

Interestingly, studies do not precisely identify an anatomic location but rather arbitrarily look for a *good spot* depending on the content [14–25]. According to recently published international guidelines (task force of ESICM) for obtaining sublingual microcirculatory measurements using the HVMs. The guidelines recommend attaining at least 3 video clips from multiple sites due to the heterogeneity of the microcirculation in the sublingual area [7]. An average of the microcirculatory values found from these multiple sublingual areas should be calculated to estimate the physiological components of the microcirculation within the tissue of interest.

An exact anatomical location within the sublingual cave for recommended microcirculatory measurements were not discussions in precise detail within the recent guideline. However, obtaining a representative measurement consisting of a mix of vessels including capillaries, venules and possibly arterioles with at least one large venule to test the existence of pressure artifacts [7] was endorsed. The guideline stated proper training and competence in using the HVM is required in order to achieve viable microcirculatory measurements for data analysis.

The present study was designed to determine a new way of sublingual microcirculation assessments based on anatomic knowledge of the sublingual cave. To date, only one study described the anatomic location for sublingual microcirculatory assessment in detail. This location was stationed on the floor of the mouth, between the lingual frenulum and the sublingual fold. Also known as the sublingual triangle [14].

We hypothesized that anatomical instructions are feasible and necessary in order to achieve adequate and consistent sublingual microcirculation measurements. We aimed to analyse the differences between non-anatomy versus anatomy guided sublingual microcirculation measurements.

## Materials and methods

### Subjects

Healthy volunteers aged between 18 and 40 years were recruited at random within the Academic Medical Center (AMC) of the University of Amsterdam. Hemodynamic parameters were measured before taking baseline microcirculatory measurements to ensure the volunteers were hemodynamically healthy. This volunteer study was performed with the approval of the Institutional Review Board of the AMC (Ref. No. W17_258). All volunteers received a full explanation of the study procedure and verbal consent was obtained.

### Hemodynamic and microcirculatory parameters

Macrohemodynamic parameters were measured from each participant in a non-invasive manner before microcirculatory measurement; these parameters were blood pressure, heart rate, peripheral capillary oxygen saturation and body temperature from the tympanic membrane. Microcirculatory density parameters were obtained using HVM in accordance with the requirements established by international guidelines [7]. Total vessel density (TVD) is the measurement of the total length of all the vessels in a specific area divided by the area itself. This parameter reflects the total amount of vessels and considered as a direct determinant of diffusion capacity [7]. Vessel density was divided into 3 different datasets, total vessel density (all vessels), small vessel density (vessels with a diameter < 20 μm) and large vessel density (vessels with a diameter > 20 μm). Although microcirculatory assessment can generate comprehensive data regarding the diffusive and conductive capacity of vessels and the blood contained within, only TVD was assessed in this study as a model representing diffusive capacity.

### Microcirculatory image acquisition

Sublingual microcirculation measurements were performed by incident dark-field (IDF) imaging, using CytoCam Video Microscope System (CytoCam, Braedius Medical, Huizen, The Netherlands). Details describing this technique are described elsewhere [3]. In brief, IDF imaging operates by epi-illuminating a region of interest using a green light (530 nm). The hemoglobin present in the red blood cells absorbs green light independent of oxygenation and the unabsorbed light is scattered into the surrounding tissue, rendering the red blood cells as dark and round globules contrasted by a light background. The generated image was observed on a computer screen and can be processed and extracted for data analysis using the manufacture’s software, CCTools (CytoCamTools Camera Manager v1.7.12, Braedius Medical, Huizen, The Netherlands) [3].

### Experimental design

**Fig. 1:**
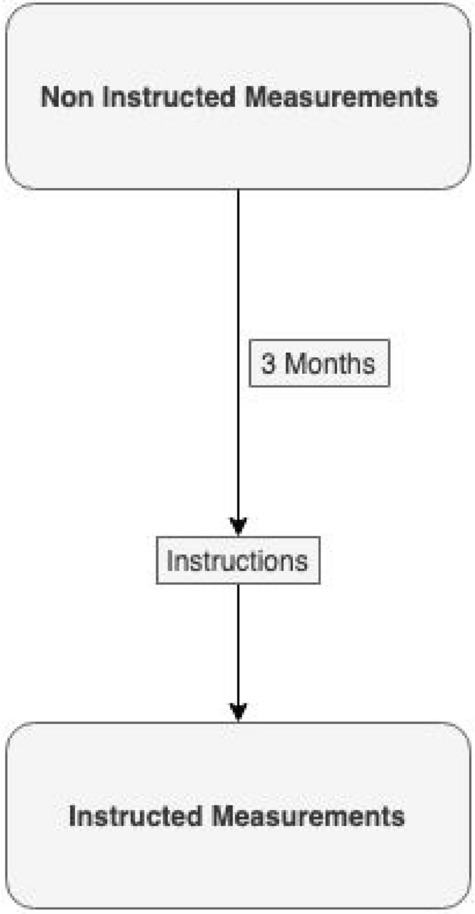
Measurement Flowchart

An inexperienced investigator (JH) performed all the measurements. The measurements were dichotomized, and repeated after 3 months. 4 video clips were obtained for each time point per volunteer (Fig. 1).

#### Instruction phase 1. Non-anatomy instructed measurements (NIM)

No anatomy specific instructions were provided to the investigator at the time of baseline measurements (T0) but adherence to established guidelines was encouraged (non-instructed measurement; NIM). The training included two parts, (i) a theoretical component in which the sublingual microcirculation was described and (ii) evaluation of image quality assessment and content criteria according to Massey et al. [26]. Briefly, a video clip was evaluated for 6 discrete criteria: illumination, duration, content, stability, pressure and focus. A score of 0 denotes optimum quality for that particular criteria and a score of 10 disqualifies the video clip as unacceptable. Scores from each criterion were totalled, and poor-quality video clips were excluded. The inexperienced observer (JH) was instructed to use a criteria table to judge the sublingual microcirculatory video images and determined which content was required for consideration as an ideal region of interest [26]. The visualisation of the region of interest can be described as a section of the microcirculation imaged without bubbles, excessive saliva containing an assortment of capillaries, venules and if possible containing arterioles. The largest vessels observed in the microcirculation are venules, and visualisation of this structure was important for judging pressure artifact induced by the operator. Imaging repetitive microvascular structures such as capillary loops were discouraged and network-based microvasculature was considered accurate [7, 26]. After the theoretical education, a practical demonstration of sublingual microcirculation imaging was shown. The observer was now considered trained on the CytoCam handling and related instrument operations (Table 1).

**Table 1:** Cytocam device operating procedure

Steps	Cytocam device operating procedure
1. Device set-up	The Cytocam imaging HVM is set-up and connected to the computer
2. Cc-tools software started	The CCTools software which is founded embedded in the computer is launched and ready for microcirculatory video capture
3. Device ready to record	CytoCam directly records video images onto the coupled computer hard disc. Ensure the green START button is active and ready to record
4. Disposable cap	The imaging probe of the CytoCam device is covered with a sterile plastic disposable cap and this alert is confirmed on the CCTools software before image capture
5. Probe placed on the ROI	The imaging probe of the CytoCam device must be placed on the region of interest (ROI). The tip of the imaging probe should gently touch the tissue surface, avoiding pressure on the microvascular system
6. Focus	Visible microvascular structures can be bought into focus through manipulation of the focus tool within CCTools After obtaining a sharp, clear image of the microcirculation within the region of interest, CytoCam must be held still for a minimum of 4 s enabling the recording of a stable microcirculation video clip
7. Video-images are stored	The video images by CytoCam are captured by the CCTools software and is set to analyze, store and catalogue the obtained data on a hard disk. The data can be exported for data analysis on dedicated software programs

#### Instruction phase 2. Anatomy instructed measurements (IM)

**Fig. 2:**
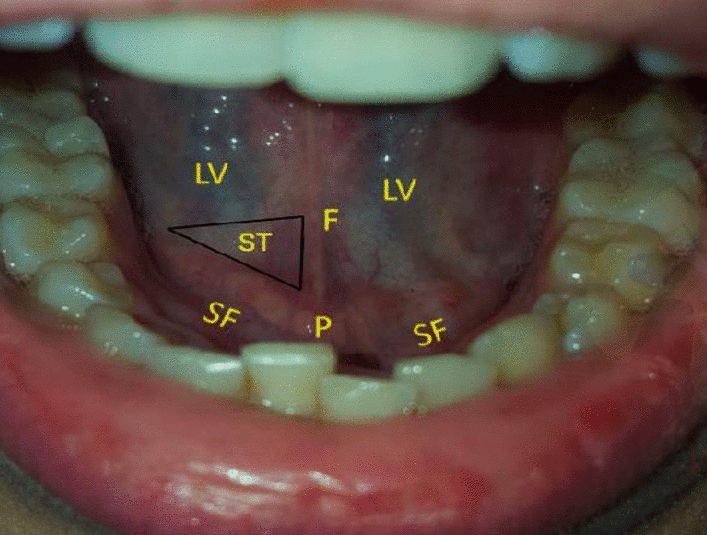
The sublingual cavity and floor of the mouth. F: lingual frenulum SF: sublingual fold, mostly salivary exit ducts (duct of Rivinus) and found bilaterally to the frenulum and extending laterally towards the inner mandible. P: sublingual papillae or caruncle, two large exit ducts (Wharton’s duct) bilaterally adjacent and at the bottom of the frenulum. ST: sublingual triangle LV: large vein

For the second set of measurements (T1), the investigator was instructed to perform microcirculation imaging guided by the anatomy of the sublingual mucosa (floor of the mouth). This specific anatomy-guided sublingual microcirculation measurement was previously published and identified as a location termed “sublingual triangle” (anatomy instructed measurement; IM) [14]. Briefly, this triangle is located bilaterally to the lingual frenulum and in between the sublingual folds. It is characterized by a good distribution of capillaries and venules forming a network-based vessel density (Fig. 2). Instructions provided to the observer consisted of identifying the aforementioned area and directly placing the tip of the CytoCam probe in the triangle.

###  Anatomy of the sublingual cave

**Fig. 3:**
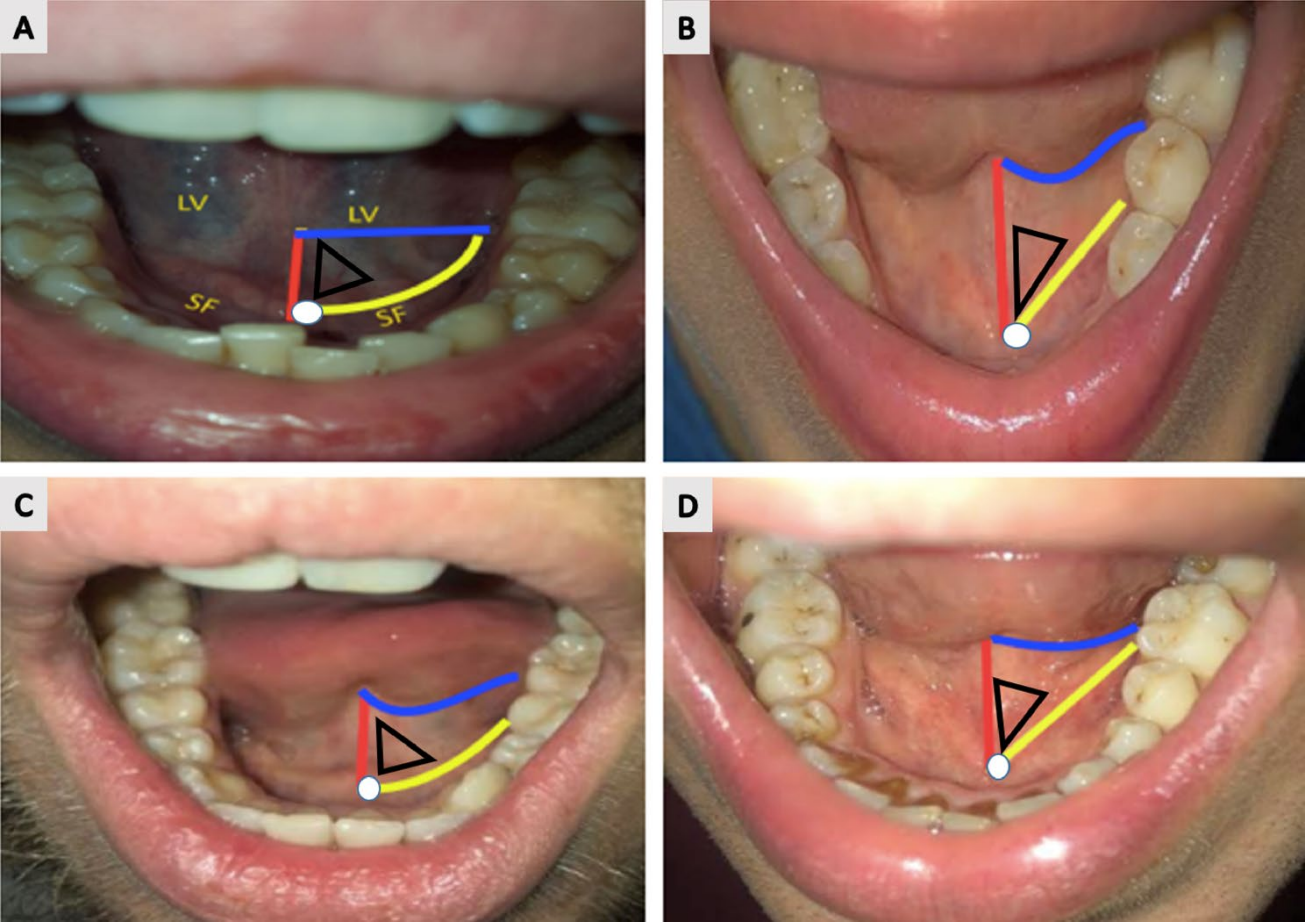
Samples of four different sublingual regions from different subjects. The acronyms and lines show all pertinent sublingual anatomic structures. LV is an area that contains large veins. In these images 3 lines in different colors denote the borders of the sublingual region of interest: the lingual frenulum (red line), the sublingual fold (SF, yellow line) and the base of the tongue (blue line). The white dot represents the sublingual papilla on the left side. The black triangle is the region of interest in between the frenulum and the sublingual fold, next to the sublingual papilla. This black triangle represents the “sublingual triangle”. Although the area between the lingual frenulum, the base of the tongue and the sublingual fold do not have the exact shape of a triangle in pictures **A**, **B** and **C**, the three sides are easily identified in the sublingual cavity. (Color figure online)

The anatomy of the sublingual cave is unique and contains three main landmarks, (i) the lingual frenulum, (ii) the sublingual papillae or caruncles and (iii) the sublingual folds bilaterally (Figs. 2 and 3). However, individual anatomic variations exist, and are characterised by variable lengths and sizes of the frenulum and sublingual folds, see Fig. 3. By using these anatomic structures in the sublingual area, the region of interest associated with microcirculation measurements can be easily identified. The papilla is considered the navigational start point for placement of Cytocam imaging probe, after identification of 3 borders marked by the lingual frenulum, sublingual fold and the base of the tongue. The papilla is considered an ideal location for placement of the probed for navigation into the sublingual triangle for microcirculation imaging. Within the triangle, a distribution of capillary networks and venules that satisfy recommended criteria for suitable microcirculation measurements and analysis may be seen [7].

###  Microvascular morphology of the different anatomical features of the sublingual cave

**Fig. 4:**
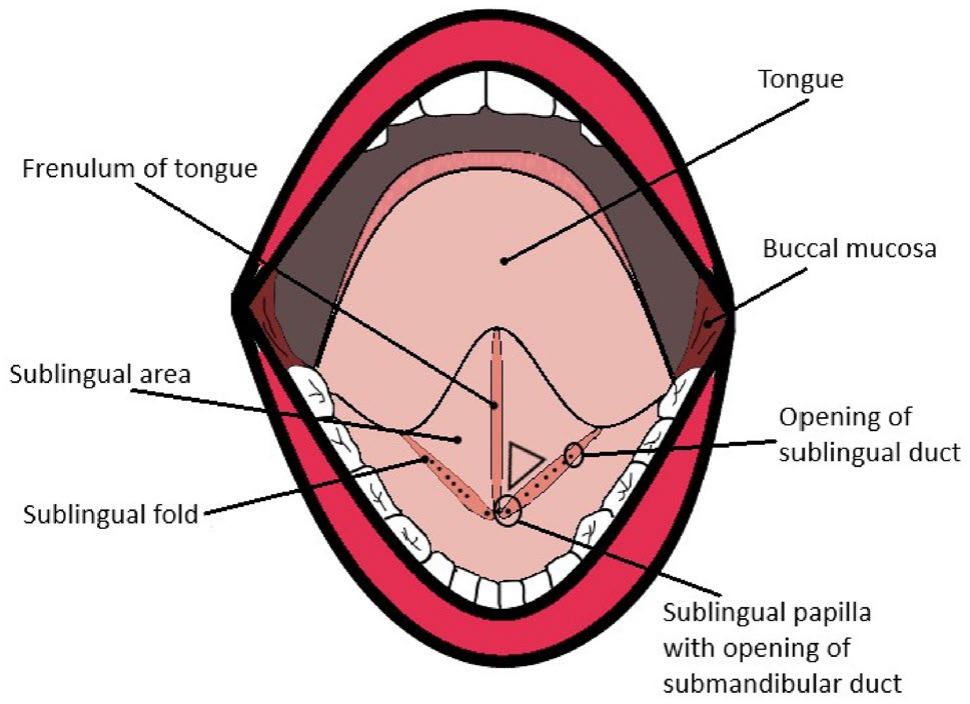
Anatomy of the sublingual cave illustrating common anatomical features of the mouth and the sublingual cave. The black triangle represents the sublingual triangle where microcirculation should be measured

After identifying the anatomic features of the sublingual cave, the associated microvascular differences and morphology was described to the observer or CytoCam operator. In Fig. 4a schematic illustration is presented of the mouth, showing the sublingual cave.

#### The frenulum of the tongue

The lingual frenulum is formed by a midline fold in a layer of the flour of mouth fascia that inserts into the middle of the inner mandibular arch. This fascia fold of the lingual frenulum is located just beneath the oral mucosa, uniting centrally together with the connective tissue on the ventral area of the tongue. The lingual frenulum is a dynamic structure that forms a diaphragm-like structure transversely situated in the floor of mouth with a membrane like appearance separating the right and left sublingual areas.

#### Sublingual triangle

**Fig. 5:**
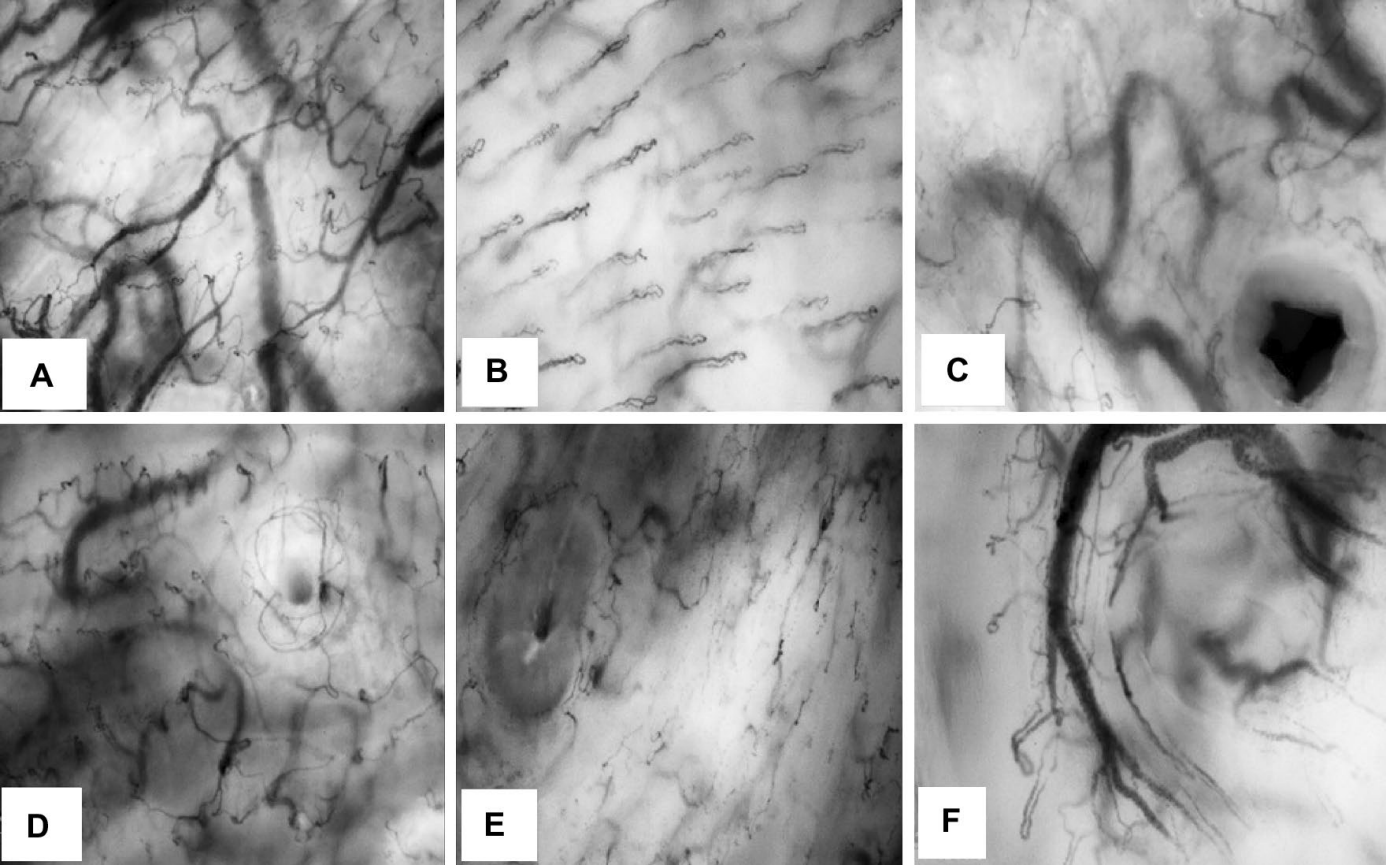
Screen shots of CytoCam video clips showing the different microcirculation morphology and architecture consistent with the different oral anatomic structures. **A** Example of sublingual microcirculation obtained from the sublingual triangle area. Sublingual area is characterized by a mix of small and large vessels. **B** Example of buccal mucosa microcirculation, the microcirculation shows arrays of repeating vessel loops. **C** Example of the microcirculation of the sublingual papilla, a black gap is surrounded by small vessels. **D** Example of the microcirculation of an opening of sublingual duct. The opening of the salivary duct is smaller than the opening duct of the submandibular duct shown at C. The small black-grey gap is surrounded by a circular-small-vessels. **E** Example of a minor salivary gland duct opening assessed at the sublingual fold. The opening shows a small black gap, surrounded by small vessels. **F** Example of the microcirculation of a mucosal papilla. At the papillary areas of the mucosa of the ventral mucosa of the tongue, plica fimbriata structure showing typical morphology with both microcirculation networks and capillary loops

The sublingual triangle is the area between the lingual frenulum, sublingual fold and the ventral base of the tongue. When the tip of the HVM device is placed next to the sublingual papilla, the apex of the sublingual triangle may be identified and will stretch ipsilaterally between the lingual frenulum and the sublingual fold (see black triangle in Fig. 4). A sample image of the microcirculation in this sublingual area is shown in Fig. 5 A.

#### Ventral mucosa of the tongue

The lining of the ventral lingual mucosa is characterized predominantly by capillary loops in its microvasculature and is structurally different to the microcirculation in the sublingual area. Vessel loops or capillary loops, are organized in an array of repeating looping structures confined to the subepithelial papillary layer just under the stratified squamous epithelial cell layer. These capillary loops also exist in all subepithelial (superficial) layers of the floor of the mouth mucosa, however due to the thin stratified squamous epithelial cell layer the focal depth of HVM device can focus easily beyond the papillary layer and onto the network-based microcirculation of reticular layer. A sample of ventral tongue mucosa capillary loops is illustrated in Fig. 5B.

#### Sublingual papilla

The sublingual papilla is a large exit duct located bilaterally at base of the lingual frenulum. The main submandibular salivary gland duct or Wharton’s duct extends anteriorly from the gland into the sublingual space of the flour of the mouth and ends in the sublingual papilla. The sublingual papilla can appear open or closed during the sublingual microcirculatory assessments and are easily differentiated from other minor (sublingual) exit duct due to its large size and shape. The microcirculation around the papilla shares a layout highly similar to the minor sublingual exit ducts and mirrors a ring-like structure (Fig. 5 C).

#### Duct of Rivinus in the sublingual fold

The floor of the mouth contains multiple salivary gland exit ducts that are responsible for secreting viscous saliva into the sublingual cavity, see Fig. 4 for the opening of these salivary ducts. The sublingual fold is fused with a folded layer of fascia on the floor of mouth and is located bilaterally in the sublingual cave. It contains the opening ducts for the sublingual glands or collectively known as the duct of Rivinus. A network of capillary structures envelopes these structures and they are embedded into the sublingual fold (Fig. 5D and E).

#### Minor mucosal folds/papilla

Along the ventral mucosa of the tongue several small mucosal extensions resembling the papilla can be found. These are known as fimbriated folds of the tongue or plica fimbriate. Essentially these structures are isolated extensions of normal mucosa that appear as vascularized protrusions consisting of both network and capillary loop microcirculation type vasculature (Fig. 5 F).

### Analysis of the sublingual images

**Fig. 6:**
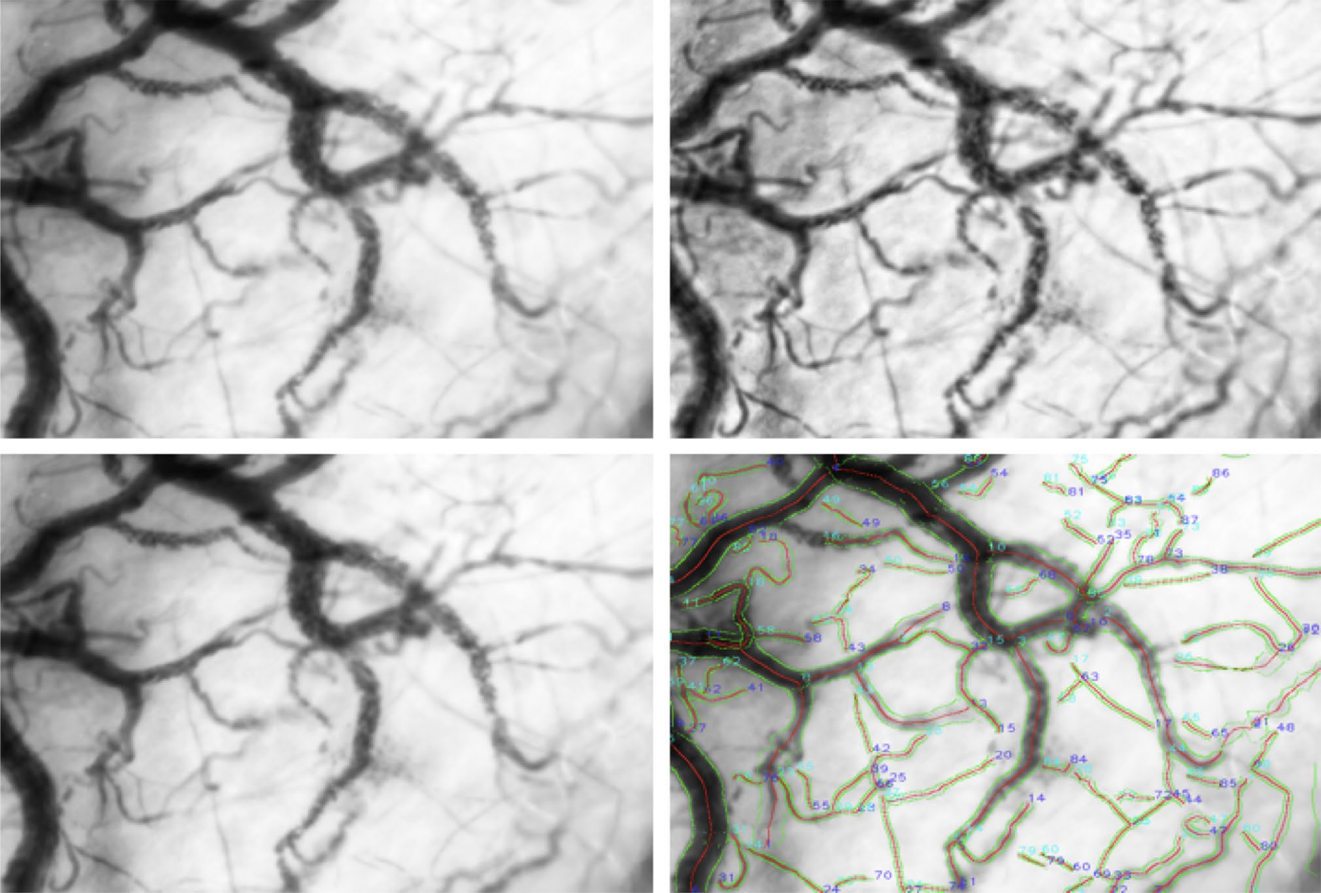
Automatic processing and analysis of microcirculatory parameters by MicroTools illustrated in four steps

After image acquisition, the video clips were exported according to manufacture instructions using h CCTools. To ensure a high-quality dataset, the acquired images were first screened and scored for quality before further analysis as proposed by Massey et al. [26]. After video clip quality assessment, images were analyzed using a newly introduced, completely automated MicroTools software [27]. A sample of the automated image processing steps is presented in Fig. 6.

### Statistical analysis

Descriptive statistics were used to present participant characteristic variables. All variables were tested for normality using the Shapiro-Wilk test. For TVD, LVD and SVD a paired t-test was used and for the comparison between diameters an unpaired t-test was used. A 2-sided p-value of < 0.05 was considered significant. A Wilcoxon signed-rank test was used to assess Massey image quality scoring. A chi-square test was used to compare the percentage of the number of salivary gland associated video clips in each group. Variables were analyzed using SPSS, version 25 for Mac (IBM Corp., Armonk, NY, USA) and GraphPad Prism 8 for Mac (GraphPad, San Diego, CA, USA).

## Results

### Volunteer characteristics and hemodynamic parameters

12 volunteers in total were enrolled in this study. Baseline characteristics of the volunteers are presented in Table 2. Three volunteers were excluded from analysis as the participants were unable to attend the second measurement (T1). As such a total of 9 volunteers were included for final analysis.

#### Volunteer characteristics at baseline

**Table 2:** Healthy Volunteer Characteristics. Data is presented as mean ± standard deviation (SD). All parameters were measured at baseline (NIM; T0). SpO_2_: peripheral capillary oxygen saturation

Age (years)	32 ± 6
Gender (male: female)	8:1
Heart rate (bpm)	67 ± 11
Systolic blood pressure (mmHg)	118 ± 8
Diastolic blood pressure (mmHg)	73 ± 7
Mean blood pressure (mmHg)	88 ± 6
SpO _2_ (%)	97 ± 2
Body temperature (ºC)	36.8 ± 0.3

#### Included video clips after quality assessment

A total of 72 video clips were recorded in total, 36 video clips during the NIM (T0) and 36 video clips after 3 months during the IM (T1). Videos were scored according to the criteria described by Massey et al. [26] in terms of content related parameters. 15 clips were excluded due to poor image quality, i.e. 8 (22%) in NIM vs. 7 (19%) in IM. Which resulted in 28 video clips retained for NIM and 29 video clips for IM further analysis.

#### Video clips containing salivary gland associated microcirculation

**Fig. 7:**
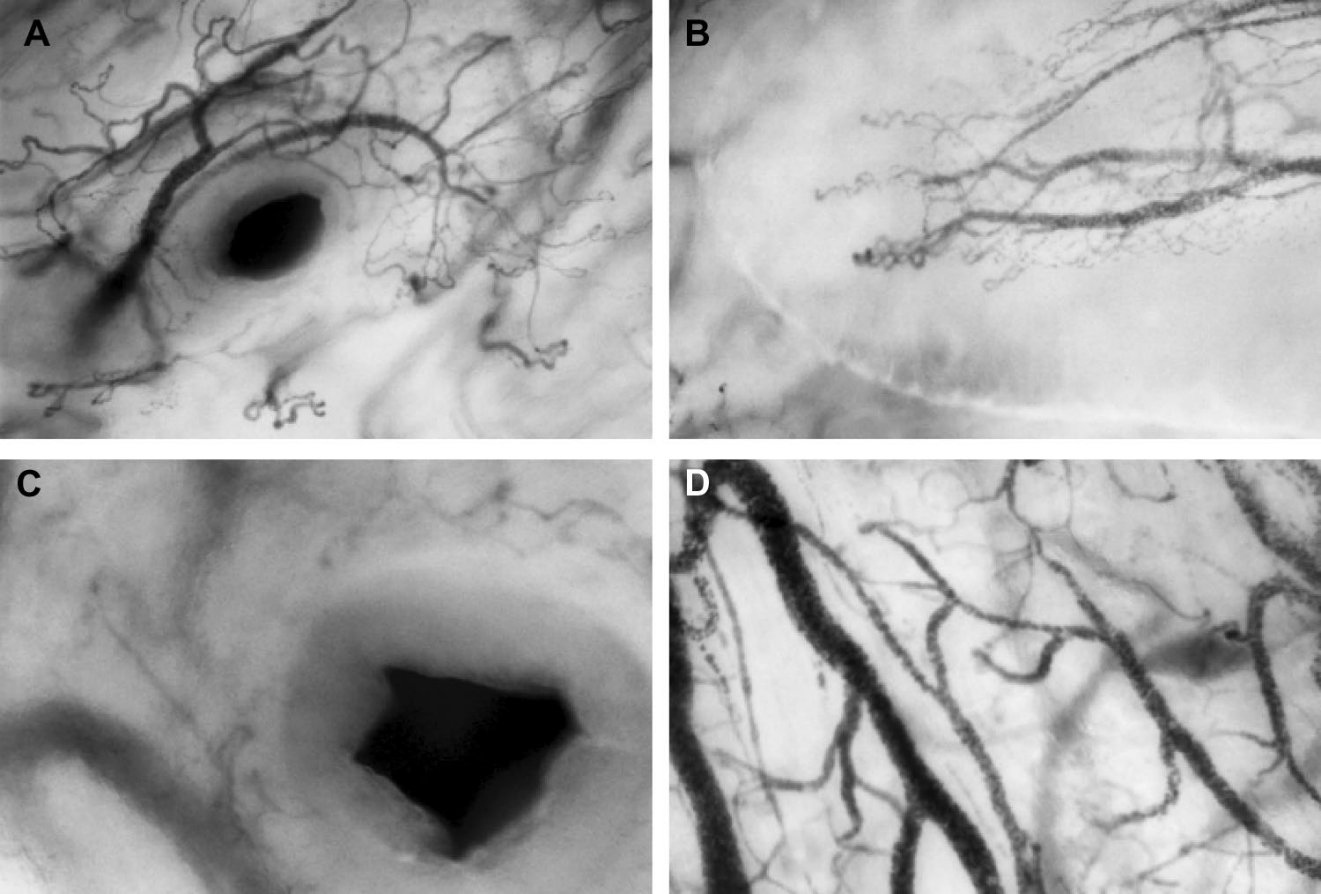
Sublingual mapping: **A** Top Left, an exit duct with circular microcirculation surrounding a black gap. **B** Top right shows a mucosal papilla of the sublingual fold with its distinctive microcirculation. **C** Bottom left shows the exit of the papilla. **D** Bottom right shows the overview of the sublingual triangle

The video clips obtained in this study showed salivary gland duct associated structures in the NIM group, whereas the presence of these structures was absent in IM measurements. The NIM group showed 6 video-clips containing salivary gland associated anatomy (6 of 28 video-clips, 21%), where the IM showed no salivary gland (0 of 29 video-clips, 0%). The number of slavery gland associated microcirculation is significantly higher in the NIM group, 21% vs. 0% with a p > 0.001 using chi-square test. Furthermore, anatomy-guided approach to measuring the sublingual microcirculation showed increased consistency in measurements, and accuracy of microcirculation features compared to NIM. Figure 7 illustrates samples of microcirculation consistent with special anatomic structures found in the sublingual region.

### Vessel diameter distribution

**Fig. 8:**
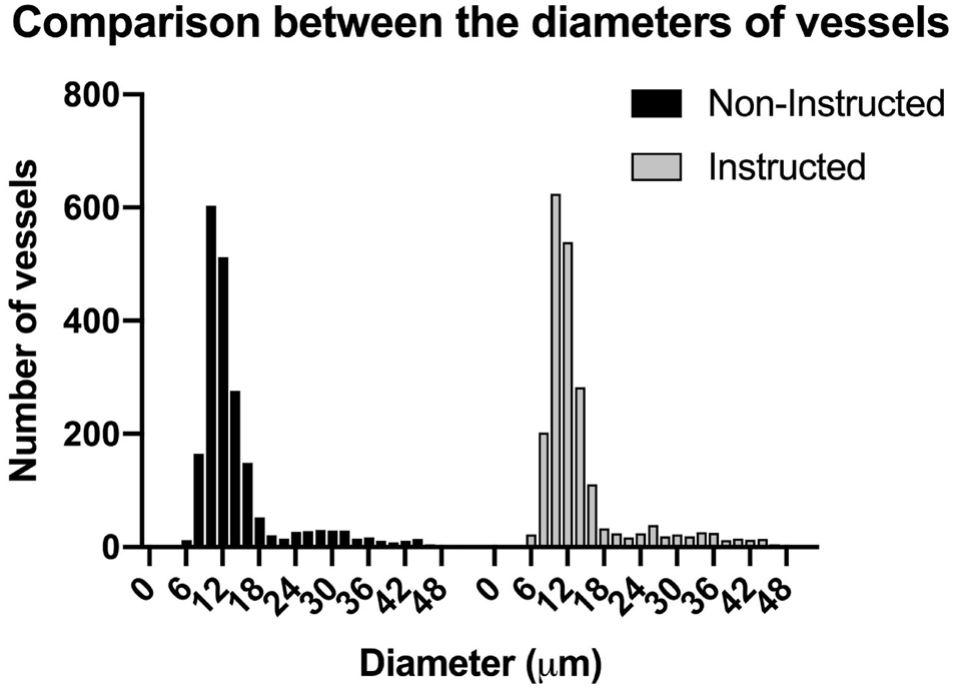
Diameter distributions of the microcirculation vessels obtained during the non-anatomy instructed (NIM; T0) and anatomy instructed measurements (IM; T1)

There was no statistical difference in average diameters of all vessels between the NIM (T0) and IM (T1) groups (14 ± 7 micrometers vs. 14 ± 8 micrometers; p = 0.58). This is shown in Fig. 8, a histogram of analysed vessels displaying a binary distribution in both groups with a larger peak depicting a concentration of capillaries and smaller peak consisting of large capillaries and venules. (Fig. 8).

### Microcirculatory vessel density parameter

**Fig. 9:**
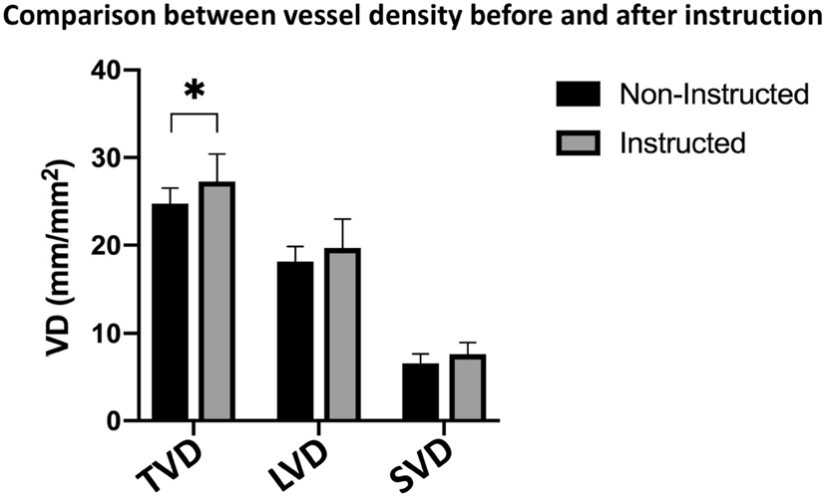
Comparison of vessel densitybetween non-anatomy instructed and anatomy instructed measurements. Total vessel density *p-value < 0.05. TVD; total vessel density, LVD; large vessel density, SVD; small vessel density

The results of TVD, LVD and SVD are shown in Fig. 9. A significant difference in TVD was found between NIM (T0) and IM (T1), 25 ± 2 vs. 27 ± 3 mm/mm^2^ (p = 0.044). However, this significant difference was not detected in LVD parameters (7 ± 1 vs. 8 ± 1 mm/mm^2^; p = 0.092) nor in SVD parameters for NIM vs. IM (18 ± 2 vs. 20 ± 3 mm/mm^2^; p = 0.103) measurements respectively.

## Discussion

The aim of this study was to demonstrate the feasibility of basic theoretical and practical training for obtaining anatomy guided microcirculation measurements. Results from microcirculation images captured in the sublingual triangle showed consistent data acquisition in relation to microvascular architecture and morphologies associated with anatomy guiding. Data derived from this area showed no salivary duct associated structures, thus reducing the possibility of including covariate microvascular structures. These findings indicate that the sublingual triangle has the potential of being a precise location for consistent microcirculation measurements since it can be easily identified and consists of the network-based microvasculature necessary for adequate analysis and interpretation of sublingual microcirculation.

Important points are to be considered in view of implementing standardized microcirculation measurements into routine clinical practice. For instance, research has reported poor intra- and interobserver reproducibility when measuring sublingual microcirculation [28]. Furthermore, large range of TVD values are in literature which may not always be attributable to underlying pathology. For the most part this could be due to a lack of data from healthy individuals spanning across several age categories for inauguration of a standard TVD index. TVD values of 2 to 20 mm/mm^2^ are reported in different patients, and their microcirculation was measured across various clinical care timepoints during treatment. For instance, at the time of hospital admission or prior to initiation of treatment [5, 29–32]. Although these results could be attributable to various confounders such as comorbidities, microcirculation examiner expertise, innate anatomic variations and lack of normal control values; few studies precisely identify an exact area of interest for microcirculation measurements associated within the sublingual region. Building a concept of sublingual triangle allows investigators to orientate and perform measurements quickly on a morphologically suitable location. Including salivary duct associated microcirculation in data analysis can artificially lower TVD values when captured during microcirculation assessment. The appearance of a duct opening in the video image takes up space within the field of view, which is otherwise necessary for robust microvascular analysis, thus resulting in lowered density parameters.

Yet, the large variations in sublingual microcirculatory density found in the literature is not understood [5, 29–32]. A possible hypothesis, could be the effect of the salivary gland associated microcirculation. However, in diseases involving the salivary glands such as Sjogren’s syndrome, targeted research can focus on examining peripheral duct microcirculation. In such cases, knowledge of the sublingual triangle and surrounding anatomic structures can be useful to facilitate targeted measurements. Orientation and a keen understanding of intraoral anatomic structures in combination with practical training is necessary in order to achieve a baseline competency level to yield viable measurements of the sublingual microcirculation. To prove the possible association between the large variations found in the literature for the microvascular density and the salivary gland associated microcirculation, there is need for more research with large sample sizes.

The current gold standard for analyzing sublingual microcirculation is by utilising a semi-automated software platform known as the Automated Vascular Analysis (AVA) software (MicroScan BV, Amsterdam, The Netherlands). However, this manner of analysis is time consuming and requires significant skill and precision on behalf of the analyser.

In the present study, a fully automated software was used to analyze the sublingual microcirculation datasets [27]. An automated analysis platform indeed comes with both advantages and disadvantages. The advantage of time reduction during the image analysis processing stage, and a potential disadvantage could be lack of precision possibly owing to the absence of user interaction. However, in a race against time to access necessary microcirculation details to potentially guide clinical decision making in critical care patients, reduced analytical time could be highly valued.

Comparison between previous TVD measurements from another study [14] and the current TVD derived from this investigation’s sublingual triangle showed remarkable agreement in outcome. It is interesting to note that, despite the lack of precisely defining the anatomic site of sublingual measurements performed in other studies, TVD comparisons appear to be similar [34–36]. The present study measured TVD in comparable healthy subjects using the same HVM device [3]. The findings of TVD in non-anatomy instructed sublingual microcirculation measurements, are in line with TVD findings obtained in 25 healthy volunteers. Nevertheless, the comparison of the TVD values between these studies and the present study, may not be entirely appropriate, as the present study has a smaller sample size and utilized a new fully automatic software for analysis.

Another important finding is the amount of video-clips, that were not included due to bad quality. These video-clips were excluded due to pressure artefacts. This implicates the fact that the observer was unexperienced, and did not have the extensive exposure and training in between the measurements. For improvement of quality we know from training courses and expert opinion, that the observers need extensive training in microcirculatory assessments to improve the quality of the measurements. This extensive training is necessary to avoid pressure artefacts. The right handling of the video-microscopy probe, and the ability to release pressure without losing the image of the microcirculation is an important skill. Thereby, is the image acquisition the most important limiting step for the clinical introduction of the video-microscopy.

There are several points that needs to be considered in regard to the study design. Firstly, one inexperienced examiner was employed to perform microcirculation assessments. This approach reduced the possibility of inconsistencies between measurements resulting from the user’s learning curve. As such, we were unable to conclusively identify any interobserver differences. Additionally, an inexperienced examiner of microcirculation closely resembles a real-life scenario in which HVM devices are used by untrained investigators. Our results, in terms of vessel distributions (small and large vessels), showed a normal distribution in the imaged sublingual mucosa. It is not known if this proposed anatomy guided measurement would lead to enhanced data quality in patients that are known to already have a higher TVD, such as in the case chronic hypertension patients.

In this study, a new fully automated microvascular analysis [27] method was used for data analysis. Imaged clips were fully blinded and processed objectively. This, in turn, reduced risk of bias affiliated with semi-automated software examination which is still considered the golden standard. Examiners were not privy the history of the microcirculation images and therefore unconscious biases may be mitigated.

Although there was an increase in SVD and LVD T0 and T1 (NIM vs. IM), these findings were not statistically significant. A possible explanation could be attributed to the small sample size of this study. Lastly, the measurements were obtained in healthy volunteers which will have limited applicability to a critical care setting. Critically ill patients are mostly ventilated, presenting limited access into the oral cavity. In addition, due to the presence of the oral tube, the sublingual cave is often clouded by saliva thickening and accumulation. Future studies should further explore the possibility of on anatomy guided sublingual measurements in diseased and ventilated patients.

In conclusion, sublingual microcirculatory measurements performed in the sublingual triangle showed morphological properties corroborating with international guidelines, and demonstrated no salivary gland associated microcirculation. We propose careful reassessment regarding sublingual microcirculation measurements to include the use of an anatomy guided strategy. Using the sublingual triangle as a foundation, the user may avoid salivary duct associated structures, and increase imaging consistency associated with clinical HVM. Finally, a higher total vessel content was identified in the anatomy instructed measurements compared with non-anatomy guided measurements. There is a need for future studies with larger sampled cohort to show density and flow parameters, as well as demonstrate reproducibility of sublingual microcirculation assessment in the sublingual triangle.
